# Detection of Alzheimer’s Disease-Related Cognitive Change With the Mobile Cognitive App Performance Platform

**DOI:** 10.1093/geroni/igaa057.2907

**Published:** 2020-12-16

**Authors:** Dawn Mechanic-Hamilton, Sean Lydon, Alexander Miller, Kimberly Halberstadter, Jacqueline Lane, Sandhitsu Das, David Wolk

**Affiliations:** University of Pennsylvania, Philadelphia, Pennsylvania, United States

## Abstract

This study investigates the psychometric properties of the mobile cognitive app performance platform (mCAPP), designed to detect memory changes associated with preclinical Alzheimer’s Disease (AD). The mCAPP memory task includes learning and matching hidden card pairs and incorporates increasing memory load, pattern separation features, and spatial memory. Participants included 30 older adults with normal cognition. They completed the mCAPP, paper and pencil neuropsychological tests and a subset completed a high-resolution structural MRI. The majority of participants found the difficulty level of the mCAPP game to be “just right”. Accuracy on the mCAPP correlated with performance on memory and executive measures, while speed of performance on the mCAPP correlated with performance on attention and executive function measures. Longer trial duration correlated with measures of the parahippocampal cortex. The relationship of mCAPP variables with molecular biomarkers, at-home and burst testing, and development of additional cognitive measures will also be discussed.

